# Mendelian randomization and colocalization analyses reveal an association between diet consumption and altered telomere length in leukocytes

**DOI:** 10.1097/MD.0000000000045825

**Published:** 2025-11-21

**Authors:** Bowen Wang, Jiawei He, Zijuan Pei, Chunhui Tao, Enfeng Song

**Affiliations:** a College of Chinese Medicine, Hubei University of Chinese Medicine, Wuhan, China; b Hubei Shizhen Laboratory, Wuhan, China; c Department of Traditional Chinese Medicine, Renmin Hospital of Wuhan University, Wuhan, China.

**Keywords:** colocalization analyses, diet consumption, Mediterranean diet, nut consumption, telomere length, wine consumption

## Abstract

Observational studies suggest dietary factors influence leukocyte telomere length (LTL), yet causality remains unproven. Using Mendelian randomization (MR) and colocalization analyses, we investigated causal relationships between 38 dietary phenotypes and LTL to clarify conflicting evidence on Mediterranean diet (MD) components (alcohol, nuts) and biological aging. Genetic instruments for dietary exposures were derived from genome-wide association studies data. Univariate MR analyses were conducted using inverse-variance weighted models, supplemented by sensitivity analyses (MR-Egger, weighted median) to address pleiotropy. Multivariable MR adjusted for correlated dietary factors, while colocalization analysis (posterior probability > 0.80) identified shared genetic variants. Reverse MR and analyses excluding pleiotropic single-nucleotide polymorphisms further validated robustness. Genetically predicted champagne/white wine and red wine intake were associated with shortened LTL, whereas nut consumption increased LTL. These associations persisted in multivariable MR (*P* < .05) and colocalization analyses, suggesting causal mechanisms independent of confounding. No other dietary phenotypes showed causal links to LTL. Sensitivity analyses confirmed minimal pleiotropic bias (MR-Egger intercept *P* > .05). This MR study provides genetic evidence that moderate alcohol consumption, including wine, accelerates telomere shortening, which contradicts previous observational claims regarding the benefits of the MD that often emphasize wine as a protective component. In contrast, nut intake aligns with dietary recommendations for anti-aging. These findings challenge the prevailing notion of the MD as a uniformly beneficial model for longevity (often promoted as a “holistic healthy pattern”) by revealing heterogeneity and even antagonistic effects among its components (alcohol vs nuts) on aging. The study underscores the need to reassess the role of alcohol in longevity-promoting diets. Public health strategies should advocate reducing alcohol consumption and increasing nut intake to mitigate the risk of age-related diseases.

## 1. Introduction

Telomeres are specialized dynamic nucleoprotein structures located at the ends of chromosomes, serving as protective caps that prevent recombination, fusion, and degradation, thus preserving the structural integrity of genetic material.^[1]^ At birth, individuals exhibit significant variability in telomere length (TL), typically ranging from several thousand to 20 thousand base pairs. Due to the “end replication problem,” where the ends of DNA are not fully replicated during cell division, TL shortens with each cell division and aging. Human telomeres lose an average of 50 to 100 base pairs per mitotic division, limiting the replicative capacity of cells.^[2]^ When telomeres reach a critical length, cellular proliferation is impeded; cells either cease dividing and become senescent, eventually undergoing “replicative death,” or undergo apoptosis regulated by genetic mechanisms. Telomere sequences, characterized by long repetitive DNA and high guanine–cytosine content, are disproportionately susceptible to oxidative stress compared to other non-telomeric sequences. Additionally, telomeric DNA appears to lack the repair mechanisms for single-strand breaks, making it more sensitive to the accumulation of such damage. TL is considered a biological clock that measures the aging process and lifespan of an organism and its cells. Leukocyte TL is highly sensitive to aging and positively correlates with healthspan, reliably reflecting the telomere status of other tissues in the body.^[3]^ Consequently, the TL of circulating cells (primarily leukocytes) has become a simple and reliable biomarker of biological aging.

Shorter telomeres are associated with a shorter lifespan and a higher risk of age-related chronic diseases. Modifying telomere attrition is feasible because the rate of telomere shortening varies significantly and is influenced by factors beyond the rate of mitotic replication, independent of chronological age.^[4]^ Telomerase, a reverse transcriptase that synthesizes telomeric DNA using its own RNA as a template, addresses the end-replication problem by extending telomeres. However, most adult cells express very low levels of this enzyme, ultimately leading to cellular senescence and death. Single-strand breaks in telomeric DNA, whether indirectly caused by DNA repair processes or directly by reactive oxygen species (ROS), are not effectively restored by repair mechanisms. Low-grade inflammatory responses are closely associated with oxidative stress, both of which are linked to telomere shortening and the onset of age-related diseases. Reducing inflammation and oxidative stress can enhance telomere stability and slow their degradation, such as through polyphenolic compounds.^[5]^ The potential to maintain normal TL through dietary intake and lifestyle interventions as a means to extend healthspan has garnered considerable interest. Increasing evidence suggests that consuming antioxidant-rich foods and/or diets high in fruits and vegetables may play a role in the maintenance of telomere biology, thereby influencing overall health and longevity.

Research indicates that human TL is positively influenced by dietary factors such as adherence to the Mediterranean diet (MD), higher intake of plant-based antioxidants, optimal vitamin and mineral nutrition, and increased consumption of ω-3 fatty acids.^[6]^ Various bioactive compounds in the MD have been shown to impact TL and regulate telomerase activity. These dietary components also reduce inflammation and oxidative stress, thereby protecting telomeres from damage. Recent literature increasingly suggests a potential link between telomere maintenance and dietary intake. However, most studies rely on questionnaires and self-reported data to assess dietary consumption patterns, raising concerns about their limitations in accurately defining dietary habits and their susceptibility to recall bias. Furthermore, the observational nature of these studies often lacks the strength to establish direct causal relationships.

To address this issue, Mendelian randomization (MR) studies use the naturally occurring random allocation of genotypes to infer the causal effect of biological exposures on outcomes, leveraging genetic variation for causal inference. Currently, there are no MR studies reporting the causal relationship between leukocyte telomere length (LTL) and dietary intake-related phenotypes. Therefore, more evidence is needed to clarify the relationship between dietary intake and LTL, with a particular emphasis on different types of dietary consumption.

In this study, we aim to comprehensively investigate the causal relationships between 38 dietary intake-related phenotypes and LTL using a 2-sample MR analysis. We hypothesize that a shared molecular mechanism may underlie the genetic correlation between dietary intake-related phenotypes and LTL. We further explore this possibility through colocalization analysis. Understanding the causal relationships between dietary intake-related phenotypes and LTL is crucial, as it has significant implications for elucidating the potential impact of dietary habits on cellular aging, tumorigenesis, and disease development.

## 2. Methods

### 2.1. Design overview

**Figure 1.**
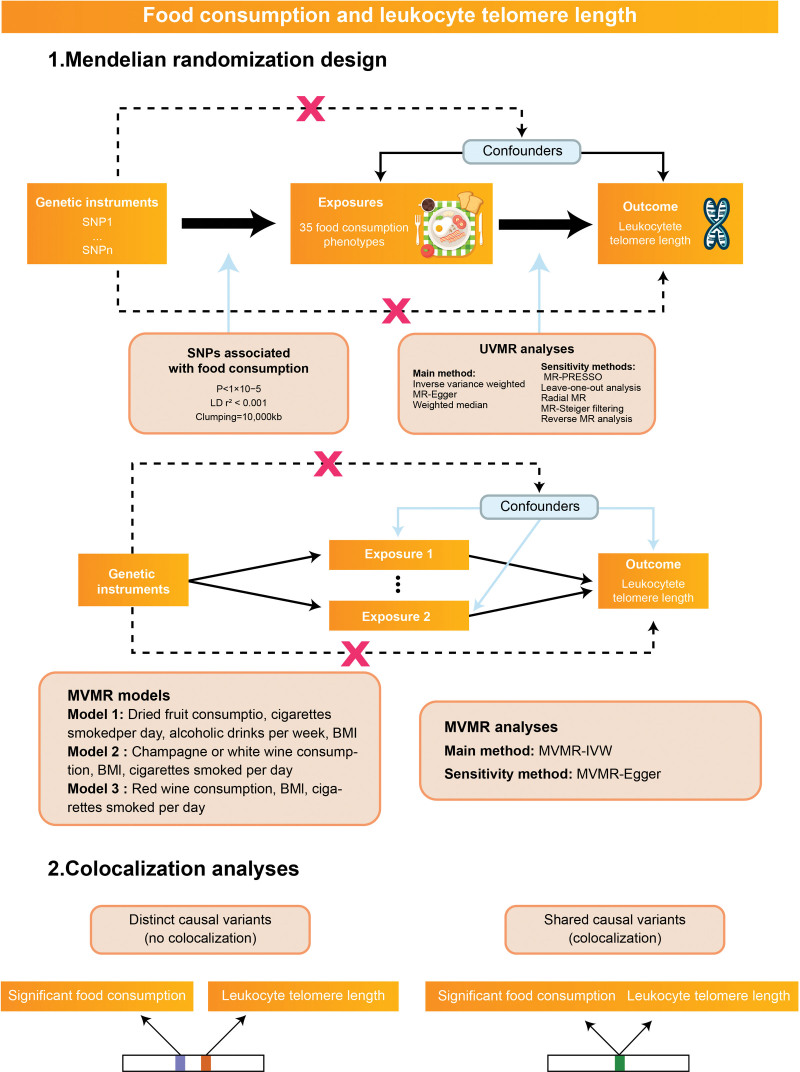
Assumptions and study design of the MR study of the associations between food consumption traits and leukocyte telomere length. IVW = inverse-variance weighted, MR-PRESSO = Mendelian randomization pleiotropy residual sum and outliers, MVMR = multivariable Mendelian randomization, SNP = single-nucleotide polymorphism, UVMR = univariable Mendelian randomization.

Figure 1 outlines the overall MR designs. Initially, we conducted univariable MR (UVMR) analysis using single-nucleotide polymorphisms (SNPs) as genetic instrumental variables (IVs) to proxy each food consumption, inferring causality with LTL. Sensitivity analyses ensured robustness of UVMR results. For significant results, multivariable MR (MVMR) analyses assessed the direct effect of food consumption on LTL, adjusting for smoking and BMI. Colocalization analysis determined if significant food consumption and LTL shared the same genetic causal variant.

### 2.2. Selection and assessment of dietary exposures

This study included 38 types of food and beverage consumption as exposures. These dietary phenotypes were selected based on the following criteria: biological relevance to mechanisms of cellular senescence (such as oxidative stress and inflammation) including key components of dietary patterns like the MD (e.g., nuts, fish, wine, fruits, vegetables) and other foods of significant public health interest (e.g., processed meats, sugar-sweetened beverages); the availability of robust genetic instruments derived from large-scale genome-wide association studies (GWAS).^[7]^ And detailed assessment within the UK Biobank cohort. Dietary habit data were obtained from the UK Biobank prospective cohort, which includes over 500,000 participants aged between 40 and 69 years at recruitment. Frequency of dietary consumption during the previous year was assessed via a touchscreen questionnaire. This questionnaire systematically collected data on a wide range of food and beverage items. To ensure robust genetic analyses and minimize potential bias from non-consumption, analyses for certain exposures (e.g., specific alcoholic beverages such as red wine and champagne/white wine, and types of coffee) were restricted to regular consumers (e.g., those consuming at least 1 alcoholic drink per week). This approach helped mitigate concerns regarding reverse causality that could arise from including large numbers of non-consumers. Genetic instrument selection for these dietary exposures followed a multi-step process. First, we identified genetic variants associated with each dietary trait from the latest and largest available GWAS summary statistics, primarily from individuals of European ancestry. Only SNPs reaching genome-wide significance (*P* < 5 × 10^−8^) were considered as candidate instruments. Second, to ensure instrument independence and avoid bias from linkage disequilibrium (LD), we clumped SNPs using a European reference genome (*r*^2^ < 0.001, clumping window = 10,000 kb). Third, we calculated the *F*-statistic for each SNP to evaluate instrument strength; only SNPs with an *F*-statistic > 10 were retained to ensure strong instruments and minimize weak instrument bias. Finally, data for each dietary trait were log-transformed where appropriate to normalize distributions prior to analysis. Table S1, Supplemental Digital Content, https://links.lww.com/MD/Q753 provides a detailed description of all 38 dietary phenotypes, including their specific assessment in the UK Biobank and sample sizes for genetic analyses. The validity of these dietary measures within the UK Biobank has been previously established.

### 2.3. Outcome

Summary statistics for LTL were derived from a GWAS meta-analysis of 472,174 individuals of European ancestry in the UK Biobank.^[8]^ LTL was measured using multiplex quantitative PCR and log-transformed and Z-standardized to minimize variability.^[9]^

### 2.4. Potential confounders

We conducted MVMR analysis to assess whether the impact of food consumption on LTL is independent of smoking, drinking, and BMI. Summary statistics for smoking and drinking were obtained from GSCAN Phase 2,^[10]^ and BMI data were acquired from the IEU database.

### 2.5. Genetic IVs selection

We identified independent SNPs (LD *r*^2^ < 0.001 within 10 Mb) with genome-wide significance (*P* < 5 × 10^−8^) as IVs for food consumption in UVMR and MVMR analyses. The LD *r*^2^ values were estimated using the 1000 Genomes Project data. Subsequently, we appraised the instrumental strength of the SNPs in UVMR by calculating the mean *F*-statistic.^[11]^

### 2.6. Statistics and reproducibility

We conducted univariate MR analyses on 35 food consumption types in relation to LTL. The inverse-variance weighted (IVW) method was used for effect size estimation, with random-effects IVW model applied upon detecting heterogeneity.^[12]^ Significant food consumptions in UVMR were further analyzed using MVMR-IVW to examine independence from smoking and BMI.^[13]^ Heterogeneity in MVMR analysis was evaluated using Cochran *Q* test and conditional *F*-statistics.^[14]^

We also conducted approximate Bayes factor localization analysis to determine if significant food consumption and LTL share a common genetic causal variant.^[15,16]^ Various MR methods, such as MR-Egger regression,^[17]^ weighted median approach,^[18]^ and MR pleiotropy residual sum and outliers (MR-PRESSO),^[19]^ were explored. Radial MR-IVW and radial MR-Egger analyses identified outliers, incorporated into further sensitivity analyze.^[20]^ MR-Steiger filtering removed genetic IVs showing reverse causation.^[21]^

Several sensitivity analyses were conducted, including leave-one-out analysis, removal of pleiotropic SNPs, and examination of associations with potential confounders using the PhenoScanner platform.^[22]^ MVMR sensitivity analyses included MVMR-Egger, MVMR-PRESSO, and MVMR-LASSO.^[23]^ We employed reverse MR to investigate the potential causal influence of LTL on significant dietary consumptions.

To address biases from partial sample overlap, we evaluated bias and type 1 error rates using an online calculator.^[24]^ In UVMR analysis, the false discovery rate (FDR) method corrected for multiple testing, setting the significance threshold at FDR-corrected *P*-value < .05. All analyses were conducted using various R software packages (RStudio, Boston ), including TwoSampleMR, MVMR, coloc, locuscomparer, MR-PRESSO, MRlap, MendelianRandomization, and RadialMR, with a 2-sided approach.

## 3. Results

Table S1, Supplemental Digital Content, https://links.lww.com/MD/Q753 provides detailed information on the genetic IVs for food consumption after LD clumping and harmonization. The mean *F*-statistics for food consumption ranged from 33.48 to 132.26, indicating a minimal chance of weak instrument bias (Table S2, Supplemental Digital Content, https://links.lww.com/MD/Q753).

### 3.1. UVMR analyses of food consumption traits on LTL

**Figure 2.**
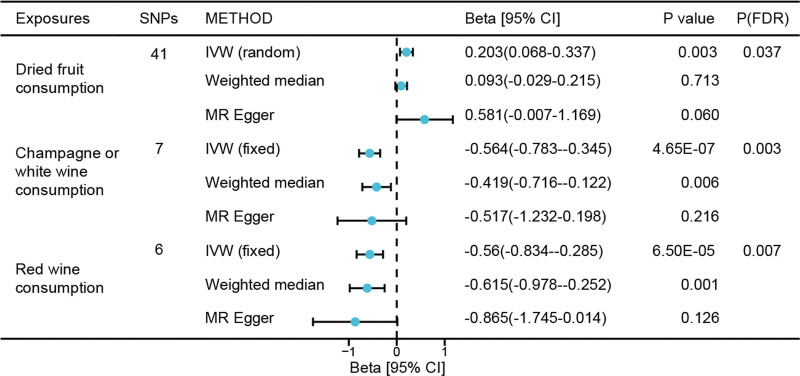
The effect of genetically determined 3 key food consumption on LTL using UVMR. The error bars indicated the 95% confidence interval corresponding to the estimates of 3 key food consumption on LTL. LTL = leukocyte telomere length, SNPs = single-nucleotide polymorphisms, *P*(FDR) = FDR-corrected *P*-value.

The IVW method showed that genetically predicted dried fruit consumption was associated with longer LTL (β [95% CI]: 0.203 [0.337–0.068]; *P* = .003, FDR-corrected *P* = .03) (Fig. 2). One-unit higher log odds of champagne or white wine consumption decreased LTL by 0.564 standard deviations (SD) (β [95% CI]: −0.564 [−0.345 to −0.783]; *P* = 4.65 × 10⁻⁷, FDR-corrected *P* = .003; Fig. 1). Similarly, 1-unit higher log odds of red wine consumption decreased LTL by 0.560 SD (β [95% CI]: −0.560 [−0.285 to −0.834]; *P* = 6.50 × 10⁻⁵, FDR-corrected *P* = .007; Fig. 1).

No causal relationships were found between other food consumption traits and LTL using the IVW method and other sensitivity MR methods (all *P* > .05; Fig. 1 and Table S3, Supplemental Digital Content, https://links.lww.com/MD/Q753). MR-Egger regression detected no horizontal pleiotropy (Table S3, Supplemental Digital Content, https://links.lww.com/MD/Q753). Although MR-PRESSO identified horizontal pleiotropy in dried fruit consumption, the results were not substantially altered after outlier correction (β [95% CI]: 0.146 [0.052–0.239]; *P* = .004; all *P* > .05 for other foods; Table S3, Supplemental Digital Content, https://links.lww.com/MD/Q753).

Scatter plots of genetic associations with LTL against food consumption are provided in Figure S1, Supplemental Digital Content, https://links.lww.com/MD/Q755. The leave-one-out analysis showed no apparent outlying SNPs, and results were stable around the expected values (Figure S2, Supplemental Digital Content, https://links.lww.com/MD/Q755).

Radial MR analyses identified between 0 to 6 outliers in the univariate MR analyses of the impact of 3 different food types on LTL (Figure S3, Supplemental Digital Content, https://links.lww.com/MD/Q755). Excluding these outliers did not significantly change the results (Table S4, Supplemental Digital Content, https://links.lww.com/MD/Q753). Additionally, MR-Steiger filtering revealed no SNPs indicative of reverse causation for these food types (Table S5, Supplemental Digital Content, https://links.lww.com/MD/Q753).

Additionally, we scanned each genetic IV in the PhenoScanner to examine whether it was associated with age-related functional impairments, chronic diseases, smoking, obesity, and heart disease. If an SNP was associated with secondary phenotypes (pleiotropic SNP), we removed it and repeated the UVMR analyses. The number of removed and remaining IVs are shown in Table S6, Supplemental Digital Content, https://links.lww.com/MD/Q753. After excluding potential pleiotropic SNPs, the relationships between genetically determined dried fruit consumption (β [95% CI]: −0.209 [−0.303 to −0.115]; *P* = .007), champagne or white wine consumption (β [95% CI]: 0.553 [0.197–0.909]; *P* = .002), and red wine consumption (β [95% CI]: 0.355 [0.008–0.703]; *P* = .04) with LTL remained consistent in both direction and significance using the IVW method (Table S6, Supplemental Digital Content, https://links.lww.com/MD/Q753).

### 3.2. Reverse MR analyses of LTL on key food consumption

The results of reverse MR analyses, including IVW, MR-Egger, weighted median, and MR-PRESSO methods, indicate no evidence of a causal relationship between LTL and the consumption of dried fruit, champagne or white wine, and red wine (all *P*-values > .05; Table S7, Supplemental Digital Content, https://links.lww.com/MD/Q753).

### 3.3. MVMR analyses of key food consumption on LTL

**Figure 3.**
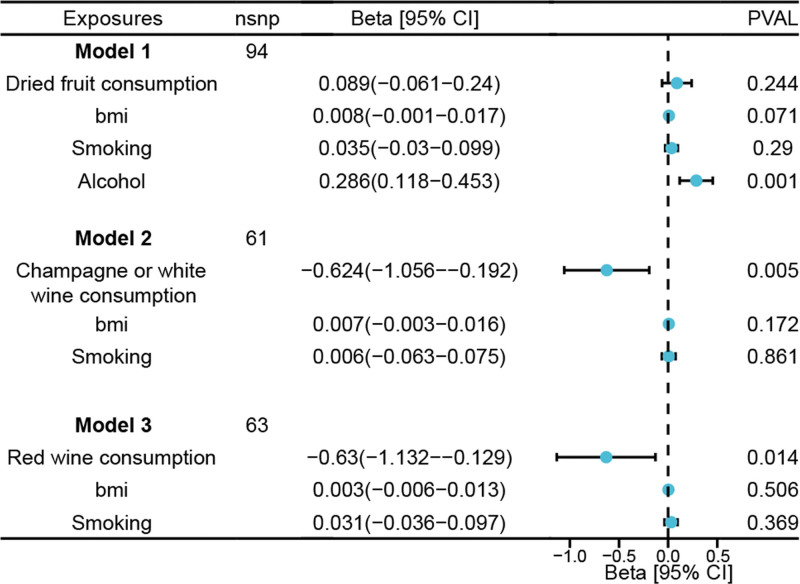
The direct effect of genetically determined 3 key food consumption on LTL using MVMR adjusted for smoking, alcohol consumption, and BMI. The error bars indicated the 95% confidence interval corresponding to the estimates of these exposures on LTL. BMI = body mass index, LTL = leukocyte telomere length, SNPs = single-nucleotide polymorphisms.

After adjusting for the number of cigarettes smoked per day and BMI, champagne or white wine consumption (β [95% CI]: −0.624 [−1.056 to −0.192]; *P* = .004) and red wine consumption (β [95% CI]: −0.63 [−1.132 to −0.129]; *P* = .01) still exhibited significant genetic predictive effects on LTL. However, after adjusting for the number of cigarettes smoked per day, alcohol consumption, and BMI, dried fruit consumption (β [95% CI]: −0.089 [−0.24 to −0.061]; *P* = .244) no longer showed a significant genetic predictive effect on LTL (Fig. 3; Tables S8–S10, Supplemental Digital Content, https://links.lww.com/MD/Q753).

### 3.4. Colocalization analyses of key food consumption with LTL

To determine whether the observed associations between dietary exposures and LTL arose from shared genetic mechanisms (such as pleiotropy) rather than LD or confounding, we conducted colocalization analyses using a Bayesian framework implemented in the coloc R package. This approach evaluates 5 competing hypotheses (H0–H4) regarding genetic association signals within a specified genomic region: H0: no association with either trait. H1/H2: association with only 1 trait. H3: association with both traits, but with distinct causal variants. H4: association with both traits, driven by a single shared causal variant. The posterior probability of H4 (PP.H4) reflects the strength of evidence for a shared causal variant; a PP.H4 exceeding 0.8 (or 80%) is generally considered strong support for colocalization in genetic epidemiology.

We applied this method to 41, 6, and 7 independent genomic regions previously linked to the consumption of dried fruit, champagne/white wine, and red wine, respectively, to evaluate their colocalization with LTL (Table S11, Supplemental Digital Content, https://links.lww.com/MD/Q753).

**Figure 4.**
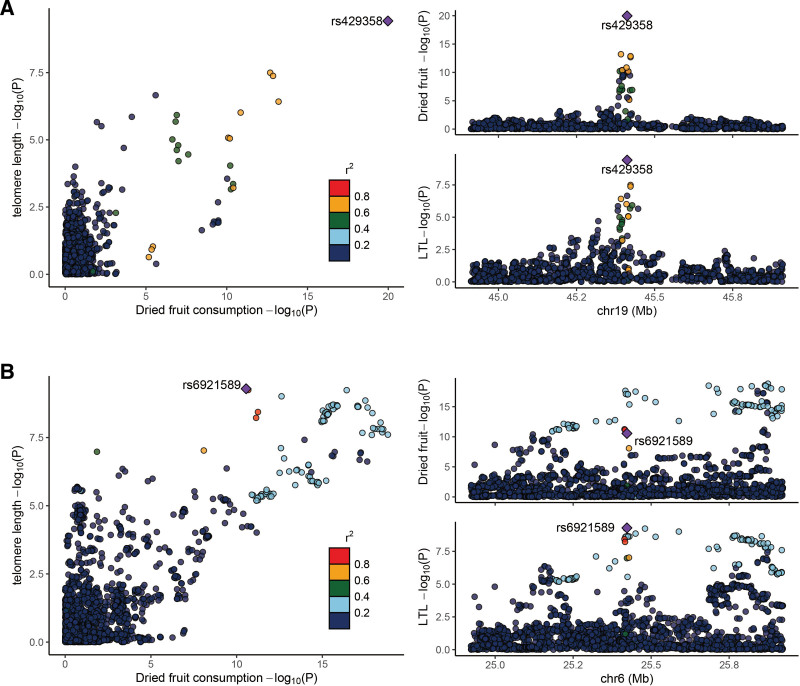
Locus comparing plots for the shared causal variant for the associations of dried fruit consumption with leukocyte telomere length. (A) Colocalization analysis results for the association between dried fruit consumption and LTL in the gene region (Chr19: rs1001611–rs9973305), which is located within ± 500 kb from rs429358. In this region, rs429358 is the lead variant identified in the GWAS of dried fruit consumption and is strongly correlated with the lead variant identified in the GWAS of LTL (LD *r*^2^ > 0.6). (B) Colocalization analysis results for the association between dried fruit consumption and LTL in the gene region (Chr3: rs1002539–rs999039), which is located within ± 500 kb from rs6921589. In this region, rs6921589 is the lead variant identified in the GWAS of dried fruit consumption and is strongly correlated with the lead variant identified in the GWAS of LTL (LD *r*^2^ > 0.6). LTL = leukocyte telomere length.

The analysis revealed a strikingly high probability (99.99%) of a shared causal variant between dried fruit consumption and LTL within a specific gene region (Fig. 4A; Table S11, Supplemental Digital Content, https://links.lww.com/MD/Q753). Moreover, within the rs6921589 region, we observed a notable colocalization signal between dried fruit consumption and LTL, with a PP.H4 of 84.95% (Fig. 4B). This value substantially exceeds the conventional threshold of 0.8, offering strong genetic evidence that the association likely stems from a shared biological mechanism (such as a variant affecting both dried fruit intake and telomere maintenance) rather than chance colocalization of independent variants.

Notably, rs429358 and rs6921589 were identified as the most probable shared causal variants within regions showing evidence of colocalization. These results bolster the MR findings by diminishing the possibility that the associations are artifacts of LD or horizontal pleiotropy, thereby reinforcing the inference of a biologically plausible causal relationship.

### 3.5. Evaluation of the impact of overlap

To assess the impact of partially overlapping participant sets between the 2 samples, we examined whether sample overlap introduced bias. The degree of overlap between individuals in the food consumption and LTL samples ranged from 6.74% to 94.41% (Table S12, Supplemental Digital Content, https://links.lww.com/MD/Q753). The type 1 error rate due to sample overlap between food consumption and LTL was controlled below 0.05, indicating minimal biases (Table S12, Supplemental Digital Content, https://links.lww.com/MD/Q753). Therefore, despite the substantial overlap between the 2 samples, significant weak instrument bias is not expected.

## 4. Discussion

In the current study, we conducted a 2-sample MR analysis to comprehensively explore the causal relationships between 38 dietary intake-related phenotypes and LTL. Our results indicate that genetically predicted self-reported nut consumption is directly associated with longer LTL, whereas genetic predisposition to alcohol intake, including champagne or white wine consumption and red wine consumption, is strongly associated with shorter LTL. Reverse MR analysis revealed no significant effect of genetically predicted LTL on nut consumption, champagne or white wine consumption, or red wine consumption. Sensitivity analyses provided further evidence supporting the potential causal relationships between nut consumption, champagne or white wine consumption, red wine consumption, and LTL, thereby reinforcing our findings. Finally, colocalization analysis identified rs429358 and rs6921589 as the most likely shared causal variants in regions showing evidence of colocalization between nut consumption and LTL, suggesting a shared genetic basis for these traits.

Modifiable lifestyle factors significantly impact overall health, with dietary patterns and food choices exerting daily effects on the human body. Dietary structure is closely linked to human lifespan, morbidity, and mortality, as exemplified by the MD.^[25]^ Numerous studies have demonstrated that TL is closely influenced by dietary factors.^[26]^ Consistent with these observational studies, our findings suggest a causal relationship between nut consumption and the attenuation of telomere attrition, potentially even promoting telomere elongation. However, contrary to most MD studies, our results indicate that consumption of champagne or white wine and red wine does not benefit TL; in fact, it is causally associated with telomere shortening.^[27]^

Telomere length is primarily influenced by oxidative stress from free radicals and reactive oxygen species released during inflammatory responses, which chemically alter guanine–cytosine base pairs and affect telomerase activity. Other influencing factors include sympathetic nervous activity, endocrine hormone levels, radiation, and chemotherapeutic drugs.^[28]^ The antioxidant and anti-inflammatory properties within dietary patterns are generally believed to be the potential link between diet, longer TL, and increased longevity.^[29]^

Despite the traditional emphasis on the potential health benefits of polyphenols in red wine within the MD,^[30,31]^ our MR analysis provides robust genetic evidence that genetically predisposed consumption of red wine and champagne/white wine is associated with telomere shortening. This finding challenges the notion that moderate wine consumption is universally beneficial for cellular aging.^[32]^ While some studies, primarily observational or in rodent models, suggest benefits from moderate red wine intake or isolated compounds like resveratrol (e.g., maintained TL in arterial cells).^[33]^ For instance, a study exploring the relationship between LTL and different alcoholic beverages in Mediterranean populations suggests regular consumption of moderate amounts of red wine and beer, rich in polyphenols, may benefit LTL without adverse effects.^[34]^ In a rodent study, rats given moderate red wine or 0.15 mg/% resveratrol maintained stable arterial cell TLs compared to those fed a standard diet.^[35]^ However, our genetic approach, which minimizes confounding, aligns with several large human studies that report a negative correlation between alcohol consumption and LTL. A case–control study involving 457 Italian men found that LTL decreased with increasing alcohol units per day.^[36]^ Similarly, increased alcohol intake in a study of postmenopausal women in the United States was associated with decreased LTL.^[37]^ A subset analysis of 499 participants from the Helsinki Businessmen Study showed a significant correlation between midlife alcohol consumption and shorter TLs in old age.^[38]^ Consistent findings were observed in a cross-sectional analysis of the large Asklepios study cohort in Belgium, encompassing 2509 individuals, where alcohol consumption showed a negative correlation with TL in both men and women.^[39]^ Conversely, some studies have found no association between alcoholic beverages and TL.^[40]^

Some studies suggest that the polyphenolic compounds in red wine are key contributors to its health benefits.^[41]^ The most notable bioactive components include resveratrol (a stilbene polyphenol), flavonoids, and tannins, which exhibit strong antioxidant properties, reduce inflammatory mediators, and offer vascular protection.^[42]^ Resveratrol, in particular, is recognized as an autophagy inducer and a calorie restriction mimetic. It activates the sirtuin family of proteins (SIRT), with SIRT1 modulating cellular processes through deacetylation of key protein targets such as NF-κB, H3-K9, H4-K16, PGC1α, and FOXO, thereby influencing DNA repair, fatty acid oxidation, and neuroendocrine regulation.^[43]^ Moreover, resveratrol has been shown to activate telomerase in human endothelial progenitor cells and breast epithelial cells, protecting telomeres through self-renewal mechanisms.^[44,45]^

Regarding the impact of ethanol on TL, evidence from large-scale observational and MR analyses indicates a significant association between high alcohol consumption and shorter LTL.^[46,47]^ In particular, a study involving 245,354 participants from the UK Biobank revealed that individuals consuming more than 29 units of alcohol per week (approximately 232 g of ethanol) exhibited significantly shorter telomeres compared to those consuming <6 units per week. This effect was more pronounced in individuals with alcohol use disorder, whose telomere shortening was equivalent to 3 to 6 years of age-related changes.^[48]^ MR analyses further supported a potential causal relationship, with genetically predicted alcohol consumption and alcohol use disorder risk associated with reduced LTL.^[49]^ The adverse effects of ethanol are primarily mediated by oxidative stress and inflammation (key mechanisms disrupting telomere integrity).^[50]^ Ethanol metabolism generates ROS and acetaldehyde, which damage DNA and reduce antioxidant capacity. Additionally, ethanol may induce inflammatory responses via pathways such as NF-κB, though these detailed mechanistic insights, while biologically plausible, may extend beyond the core scope of our dietary-focused MR findings.^[51]^ Ethanol metabolism generates ROS and acetaldehyde, which damage DNA and reduce antioxidant capacity. Additionally, ethanol may induce inflammatory responses via pathways such as NF-κB, though these detailed mechanistic insights, while biologically plausible, may extend beyond the core scope of our dietary-focused MR findings.^[52]^

Analyzing the impact of nut consumption on telomere health across 8 clinical observational studies and 1 randomized controlled trial yielded conflicting conclusions.^[32]^ In the context of the clinical trial (Walnuts and Healthy Aging Study), the effect of walnut consumption on TL was evaluated. After 2 years, participants in the control group following a regular diet without walnuts exhibited significantly shorter telomeres compared to those in the experimental group supplementing 30 to 60 grams of walnuts daily.^[53]^ Among the 8 cross-sectional studies assessing the relationship between nut intake and TL, only 3 studies demonstrated a positive correlation after adjusting for multiple confounding factors.^[54–56]^ The NHANES study involving 5582 healthy American adults^[57]^ and the Korean Genome and Epidemiology Study in a Korean population^[58]^ both found an association between nut consumption and longer telomeres. However, 2 studies on the MD and TL, the Nurses’ Health Study in the UK^[59]^ and the Washington Heights-Inwood Community Aging Project in the US,^[60]^ found no such association. In contrast, a study on food, dietary patterns, occupational level, and LTL in an Iranian population revealed a negative correlation between nut consumption and the T/S ratio [telomere (T) to single-copy gene (S) sequence] across all occupational categories examined.^[61]^

Dried fruits are rich in antioxidants such as vitamins E and C, carotenoids, flavonoids, and polyphenolic compounds, which have potent antioxidant properties and play a role in neutralizing free radicals generated by oxidative stress. Additionally, various phytochemicals found in dried fruits, including ω-3 fatty acids, polyphenols, flavonoids, proanthocyanidins, ellagitannins, and catechins, can competitively inhibit pro-inflammatory precursors, suppress the production of inflammatory mediators, influence cell membrane structure and function, and modulate inflammation-related signaling pathways such as NF-κB, PPARγ, and JAK/STAT, thereby exerting anti-inflammatory effects.^[62]^ The dietary fiber, healthy fats, antioxidants, and various nutrients in dried fruits also significantly regulate gut microbiota, reduce intestinal inflammation, influence immune system development and function, improve the gut microenvironment, and affect TL. Nuts are abundant in protein, vitamins, minerals, dietary fiber, and healthy fats, all of which are crucial for cellular metabolism and function. These nutrients regulate the normal expression and activity of telomerase by affecting the cell cycle and proliferation, thereby maintaining telomere stability.^[63]^

In the field of nutrition, a holistic dietary approach generally offers greater benefits than the consumption of single food items or selective food combinations. Increasing evidence suggests that the health benefits of foods arise from the synergistic effects of their complex matrices, which include a variety of nutrients and phytochemicals, rather than from isolated components alone.^[64]^ Studies have also linked smoking, alcohol consumption (including champagne or white wine, and red wine), and BMI with nut intake. Even after adjusting for the effects of smoking and BMI, alcohol consumption remains associated with shorter TL. Therefore, our research underscores the importance of maintaining a balanced diet, such as the Mediterranean or Oriental dietary patterns, while highlighting that alcohol consumption has a negative impact on promoting healthy aging.^[65]^

Colocalization analysis has identified that the genetic variants rs429358 and rs6921589 are shared between nut consumption and LTL. The SNP rs429358, which influences APOE gene expression, is located in exon 4 of the APOE gene within the chromosome 19q13.32 region.^[66]^ The APOE protein plays crucial roles in lipid metabolism, cholesterol transport, and the development and repair of the nervous system.^[67]^ Numerous studies have reported associations between rs429358 and various diseases such as diabetes, hyperlipidemia, Alzheimer disease, COVID-19, myocardial infarction, and laryngeal squamous cell carcinoma. These findings support the potential molecular mechanisms linking this genetic variation with TL, primarily involving APOE serum levels and gene polymorphism.^[68,69]^ Another pathogenic genetic variant, rs6921589, is located on the long arm (q arm) of chromosome 12 within the 12q13.3 region. This variant is commonly associated with cardiovascular disease risk and has significant links to multiple lipid metabolism indicators (CHOL, LDL-C, HDL-C). However, the specific mechanisms underlying these associations remain unclear and require further investigation.^[70]^

Our MR and colocalization analyses provide robust evidence supporting a causal effect of specific dietary factors on LTL.^[71]^ We identified that increased consumption of nuts exerts a protective effect on LTL, whereas intake of champagne or white wine and red wine is associated with telomere shortening. Crucially, reverse MR analysis did not support causality in the opposite direction. Furthermore, colocalization analysis indicated shared genetic mechanisms between nut consumption and LTL, suggesting a potential biological pathway beyond mere association.^[58]^ These findings help resolve longstanding ambiguities in nutritional epidemiology. For instance, although numerous observational studies have reported inconsistent associations between meat, coffee, and tea consumption with LTL,^[60,72–75]^ our MR analysis found no statistically significant evidence for a causal relationship. The discrepancies in earlier literature may largely stem from inherent limitations of observational designs, such as measurement error, residual confounding, and complex food matrix interactions.^[76]^

The implications of our work are noteworthy. Given that telomere attrition is a hallmark of biological aging and a risk factor for age-related diseases, our results suggest dietary modifications (such as promoting nut intake and moderating alcohol consumption) could offer a viable strategy for slowing cellular aging and maintaining health. However, neither has shown a statistically significant causal relationship with LTL.^[77]^ Therefore, the practical implication of our study is not to discard the MD, but to refine its implementation for promoting longevity. Public health guidelines advocating the Mediterranean pattern should place greater emphasis on increasing nut consumption while recommending caution regarding alcohol intake. Even within this healthy dietary pattern, limiting alcohol (including red wine) could be a prudent strategy to mitigate telomere shortening and cellular aging.

Notwithstanding the robust genetic evidence provided by our MR analysis, several limitations must be acknowledged. First, potential sample overlap between exposure (dietary traits) and outcome (LTL) GWAS data from public databases might introduce inflation in type I error rates. We mitigated this concern by implementing stringent statistical controls (e.g., strict significance thresholds of *P* < 5 × 10^−8^ for IV selection) and employing the MRlap validation method, an approach supported by recent simulations in large cohorts with overlapping samples.^[78]^ Second, although we conducted extensive sensitivity analyses (including MR-Egger, weighted median, and MR-PRESSO) under different pleiotropy assumptions and yielded consistent results, we cannot entirely rule out undetected horizontal pleiotropy, which remains an inherent challenge in MR design. Third, and critically, our study was confined to individuals of European ancestry. This restriction significantly limits the generalizability of our findings to non-European populations (e.g., African, Asian, or Hispanic ancestries). Genetic architecture, LD patterns, and allele frequencies of IVs can vary substantially across ethnicities. Consequently, the causal estimates we reported for diet–LTL relationships may not be directly transferable to other genetic backgrounds. Future investigations utilizing well-powered GWAS from diverse populations are urgently needed to validate and extend our findings. Fourth, our MR analysis operated under a linearity assumption, exploring the average causal effect of dietary exposures on LTL across the entire distribution of genetic liability. This framework does not capture potential nonlinear or threshold effects. For instance, the relationship between alcohol intake and LTL might follow a J-shaped or U-shaped curve, which could be elucidated in future work using nonlinear MR methods (e.g., piecewise MR or methods utilizing fractional polynomials) or through the analysis of individual-level data, should it become available.^[79]^ Finally, TL was assessed only in leukocytes. While LTL is a widely used biomarker of systemic aging, its dynamics and potential causal relationships with dietary factors in other tissues (e.g., brain, liver) or cell types remain unexplored and warrant future investigation.

In conclusion, leveraging a comprehensive genetic approach, our work underscores the potential of dietary interventions (such as increasing nut intake and moderating alcohol consumption) in attenuating biological aging. Future studies should prioritize multi-ancestry cohorts, individual-level data for nonlinear modeling, and mechanistic research on telomere-related genetic pathways influenced by diet.

## Author contributions

**Conceptualization:** Bowen Wang, Jiawei He, Zijuan Pei.

**Data curation:** Jiawei He, Zijuan Pei.

**Formal analysis:** Zijuan Pei.

**Methodology:** Zijuan Pei.

**Project administration:** Chunhui Tao, Enfeng Song.

**Resources:** Chunhui Tao.

**Software:** Chunhui Tao.

**Supervision:** Enfeng Song.

**Writing – original draft:** Bowen Wang, Jiawei He, Zijuan Pei.

**Writing – review & editing:** Bowen Wang, Enfeng Song.

## Supplementary Material




