# Supplementary material for: Mendelian randomization and colocalization analyses reveal an association between diet consumption and altered telomere length in leukocytes
Source: Medicine (Baltimore). 2025 Nov 21;104(47):e45825. doi: 10.1097/MD.0000000000045825 (PMC12643740; doi:10.1097/MD.0000000000045825)

**figure S1 Scatter plot of genetic association with LTL against associations with 3 food consumption traits.**

Abbreviations: LTL, leukocyte telomere length; SD, standard derivation.

The associations of A. Champagne or white wine consumption, B. Dried fruit consumption, C.Red wine consumption.

Scatters showed associations of SNPs with3 food consumption traits and LTL.


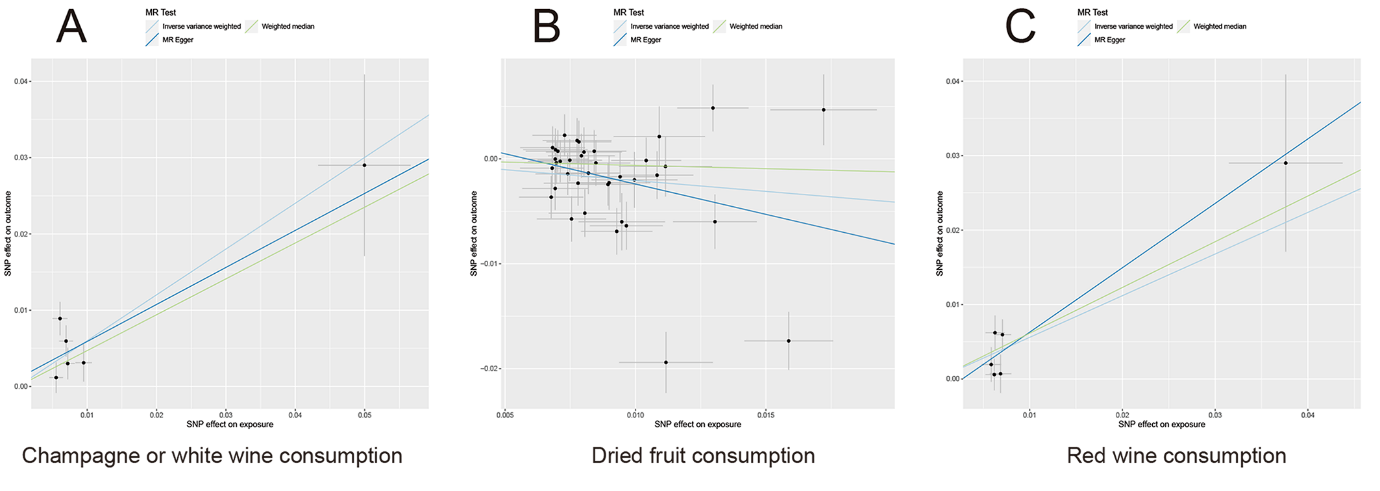


**Figure S2 Plots of leave-one-out analyses for MR analyses of 3 food consumption traits on LTL.**

Abbreviations: LTL, leukocyte telomere length;

The red lines represent the relationships of A. Champagne or white wine consumption, B. Dried fruit consumption, C. Red wine consumption.

The black line is the deviation of the 95% confidence interval corresponding to the estimate of the SNPs.


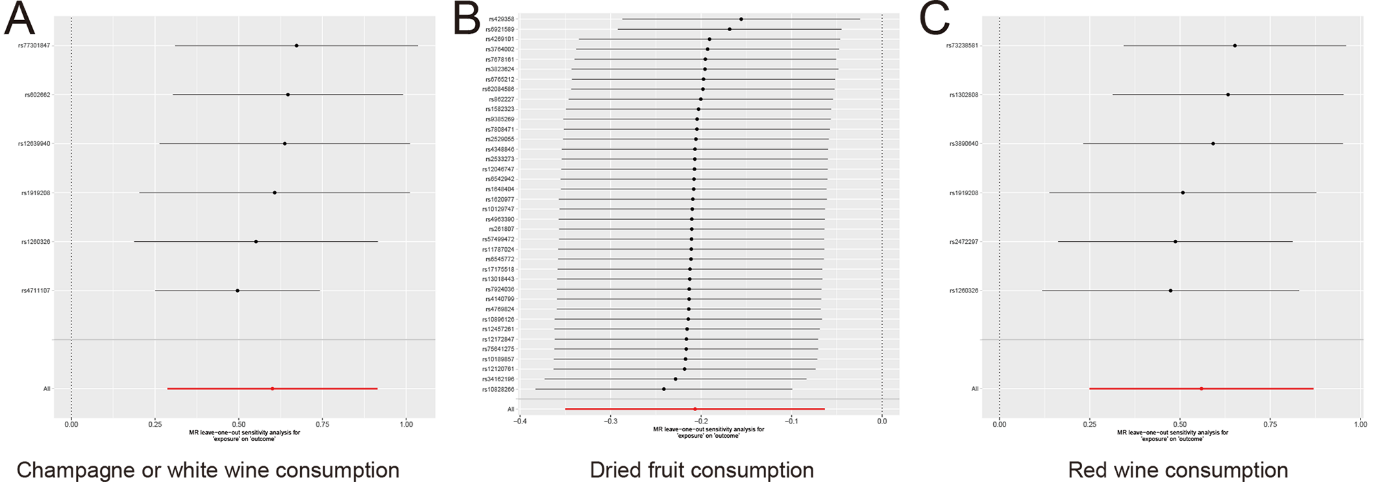


**Figure S3 Radial Mendelian randomization plots of 3 food consumption traits on LTL.**

**A**bbreviations: LTL, leukocyte telomere length; IVW, inverse-variance weighted

The figure showed radial plots of A. Champagne or white wine consumption, B. Dried fruit consumption, C. Red wine consumption.

Radial MR method identify outliers with the most weight in the MR analysis and the largest contribution to Cochran’s

Q statistic (for radial IVW) or Rucker’s Q statistic (for radial Egger) for heterogeneity, which may then be removed and the data re-analyzed. Radial curve displays the ratio estimate for the outliers (identified using Radial MR), as well as the Radial IVW (in blue) and Radial MR-Egger regression (in orange).


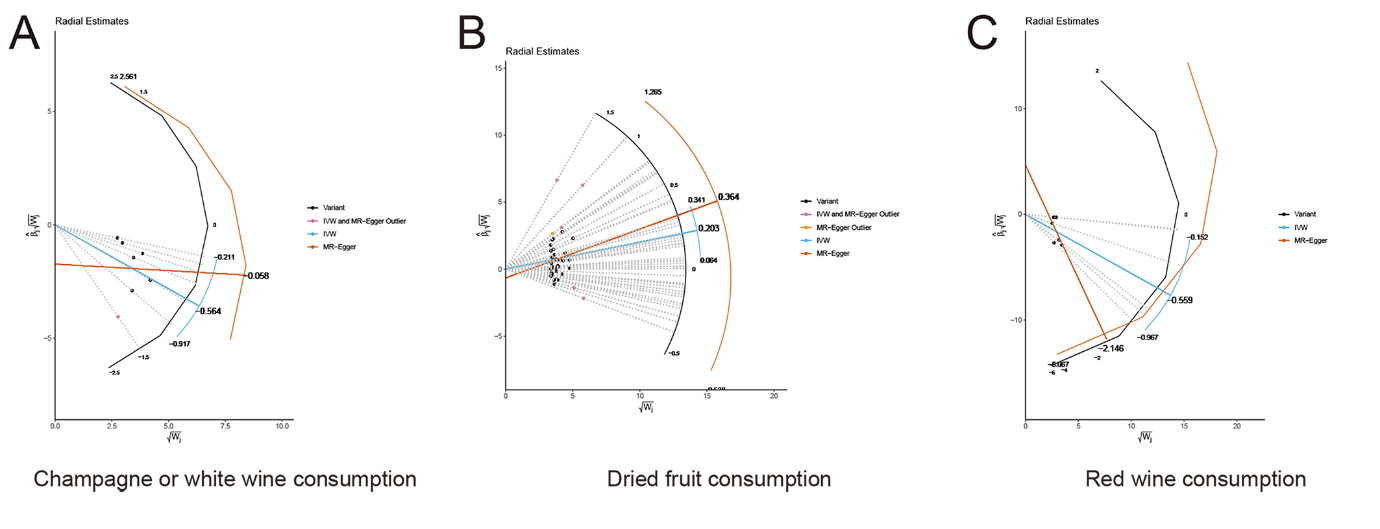

Supplement: Supplementary file 2 [file medi-104-e45825-s002.docx]
